# Absence of W Chromosome in Psychidae Moths and Implications for the Theory of Sex Chromosome Evolution in Lepidoptera

**DOI:** 10.3390/genes10121016

**Published:** 2019-12-05

**Authors:** Martina Hejníčková, Petr Koutecký, Pavel Potocký, Irena Provazníková, Anna Voleníková, Martina Dalíková, Sander Visser, František Marec, Magda Zrzavá

**Affiliations:** 1 Biology Centre of the Czech Academy of Sciences, Institute of Entomology, 37005 České Budějovice, Czech Republic; 2 Faculty of Science, University of South Bohemia, 37005 České Budějovice, Czech Republic

**Keywords:** Psychidae, Lepidoptera, sex chromosome, evolution, W chromosome, Z chromosome, genome size, sex chromatin, comparative genomic hybridization, flow cytometry

## Abstract

Moths and butterflies (Lepidoptera) are the largest group with heterogametic females. Although the ancestral sex chromosome system is probably Z0/ZZ, most lepidopteran species have the W chromosome. When and how the W chromosome arose remains elusive. Existing hypotheses place the W origin either at the common ancestor of Ditrysia and Tischeriidae, or prefer independent origins of W chromosomes in these two groups. Due to their phylogenetic position at the base of Ditrysia, bagworms (Psychidae) play an important role in investigating the W chromosome origin. Therefore, we examined the W chromosome status in three Psychidae species, namely *Proutia betulina*, *Taleporia tubulosa*, and *Diplodoma laichartingella*, using both classical and molecular cytogenetic methods such as sex chromatin assay, comparative genomic hybridization (CGH), and male vs. female genome size comparison by flow cytometry. In females of all three species, no sex chromatin was found, no female-specific chromosome regions were revealed by CGH, and a Z-chromosome univalent was observed in pachytene oocytes. In addition, the genome size of females was significantly smaller than males. Overall, our study provides strong evidence for the absence of the W chromosome in Psychidae, thus supporting the hypothesis of two independent W chromosome origins in Tischeriidae and in advanced Ditrysia.

## 1. Introduction

Moths and butterflies (Lepidoptera) are among the most species-rich groups of insects and represent the largest animal taxon with heterogametic females. Most lepidopteran species have a WZ/ZZ (♀/♂) sex chromosome constitution, but some species lack the W chromosome and have a Z0/ZZ (♀/♂) constitution, either as an ancestral sex chromosome system or as a result of a secondary loss of the W chromosome [1]. Generally, the lepidopteran Z and W chromosomes, though often similar in size, differ greatly in their composition. The Z chromosome resembles autosomes and contains many genes. Multiple studies have shown a highly conserved synteny of Z-linked genes between species across the phylogenetic tree of Lepidoptera [2,3,4,5]. In contrast, the W chromosome is largely composed of repetitive sequences and is partially or completely degenerated, possibly as a result of the absence of recombination in lepidopteran females [6]. Genetic erosion and accumulation of repetitive sequences lead to heterochromatinization of the W chromosome, multiple copies of which form a conspicuous spherical body called sex chromatin in somatic polyploid nuclei [1]. The presence of this sex chromatin body has been used as a simple, though not entirely reliable, assay to determine the presence of the W chromosome in particular species [7,8,9,10].

Female heterogamety has most probably evolved in the common ancestor of caddisflies (Trichoptera) and Lepidoptera. Since neither caddisflies nor basal moths have a W chromosome, it is believed that Z0/ZZ is the ancestral sex chromosome constitution for these sister clades [9,11]. Apparently, the W chromosome is present in the majority of lepidopteran species studied; although it should be noted that the amount of available cytogenetic data is still scarce in some groups [1]. Nevertheless, the origin of the W chromosome remains elusive. There are several hypotheses about when and how the W chromosome arose [5,11,12]. The generally accepted hypothesis of Lukhtanov [12] assumed that the W chromosome evolved in a common ancestor of Tischeriidae (Tischeriina in the original paper), a small group of leaf-mining moths, and Ditrysia, an evolutionary younger, multidiverse group comprising 98% of lepidopteran species. This assumption was mainly based on indirect evidence (such as sex chromatin absence) from taxa which had branched off earlier. However, neither this hypothesis nor the following reviews [1,6,13] considered the available data in bagworms (Psychidae), one of the most basal families of Ditrysia. In several species of bagworms, odd chromosome numbers were reported [14,15,16,17,18], possibly suggesting the absence of the W chromosome.

Recently, we investigated sex chromosomes in representatives of other basal ditrysian families, namely Gracillariidae, Plutellidae, and Tineidae, and found that the common clothes moth, *Tineola bisselliella* from the last family, lacks the W chromosome [5]. This finding, combined with odd chromosome numbers in yet more basal bagworms, casted doubts on the single W chromosome origin and led to the postulation of an alternative hypothesis of two independent origins of the W chromosome: one in Tischeriidae and one within Ditrysia after Psychidae and Tineidae branched off [5]. The odd chromosome number in females, however, does not necessarily exclude the W chromosome presence. For example, a W chromosome-autosome fusion can lead to the neo-WZ_1_Z_2_ chromosome system, which also results in an odd chromosome number in females and even number in males (see [6,19,20,21]). Nevertheless, the potential absence of the W chromosome in Psychidae is currently based solely on unequal chromosome numbers between females and males.

Considering the importance of basal families for understanding the origin of the W chromosome in Lepidoptera, we performed a detailed analysis of karyotypes and sex chromosomes in representatives of Psychidae to clarify the presence or absence of the W chromosome in this group. Implications of our findings for hypotheses on the evolution of sex chromosomes in basal moths are discussed.

## 2. Materials and Methods

We investigated three species of bagworms, namely *Proutia betulina* (Psychinae), *Taleporia tubulosa* (Taleporinae), and *Diplodoma laichartingella* (Naryciinae). Male and female larvae of Psychidae were collected in deciduous woods surrounding České Budějovice, Czechia. Larvae of the penultimate instar were found mostly on beech trunks in the period between March and June 2015–2019 and determined according to common morphological features (e.g., shape of the bag). All specimens were kept in a plastic box with moistened vegetation at a stable temperature of 4 °C and dissected within one week; residual tissues were immediately frozen in liquid nitrogen and stored at −20 °C until further use. As a control species for sex chromatin assay and flow cytometry for *P. betulina,* we used a laboratory wild-type strain WT-C of the Mediterranean flour moth, *Ephestia kuehniella* (Pyralidae), kept at the Institute of Entomology BC CAS, České Budějovice (see [22]). As a control species for the flow cytometry of *T. tubulosa*, we used a *Drosophila melanogaster* strain with white eye mutation [23] to avoid potential difficulties with the eye pigments.

### 2.1. Chromosome and Polyploid Nuclei Preparations

Meiotic chromosomes were obtained from larval gonads, mitotic chromosomes from larval gonads and brains, and in male larvae also from wing imaginal discs. Dissections were performed in physiological solution according to Glaser [24] with modifications described in Dalíková et al. [25]. Brains, wing imaginal discs, and male gonads were hypotonized in 75 mM KCl for 8 min and fixed in Carnoy fixative (6:3:1 ethanol, chloroform, acetic acid) for 15 min. Female gonads were fixed immediately after dissection in order to preserve the heterochromatin pattern of the W chromosome, if present. Fixed tissues were macerated and spread in a drop of 60% acetic acid on the slide at 45 °C using a hot plate. Preparations were dehydrated in an ethanol series (70%, 80% and 100%, 30 s each), air-dried, and stored at −20 °C until further use. Preparations used for simple staining with DAPI (4′,6-diamidino-2-phenylindole; Sigma-Aldrich, St. Louis, MO, USA) were mounted in antifade based on DABCO (1,4-diazabicyclo(2.2.2)-octane; Sigma-Aldrich) containing 0.5 μg/mL DAPI and sealed with nail polish.

For the sex chromatin assay, polyploid interphase nuclei were prepared from Malpighian tubules of the last instar larvae as described in Mediouni et al. [26]. Tubules were dissected in physiological solution, briefly fixed on a slide with Carnoy fixative, and stained with 1.25% lactic acetic orcein for 3–5 min. Each preparation was then covered with a cover slip and redundant dye was drained using filter paper.

### 2.2. DNA Extraction

Genomic DNA (gDNA) was extracted from larvae stored at −20 °C by NucleoSpin DNA Insect kit (Macherey-Nagel, Düren, Germany) with the following modifications: the tissue was initially crushed by pestles in 1.5 mL microcentrifuge tubes with 100 µL of Elution buffer BE to maximize DNA yield and then transferred to a NucleoSpin Bead Tube type D (provided by the producer). The next steps were performed according to the manufacturer’s instructions. Final concentrations of the extracted DNA were measured on a Qubit 3.0 Fluorometer (Invitrogen, Carlsbad, CA, USA).

### 2.3. Comparative Genomic Hybridization

Comparative genomic hybridization (CGH) was performed according to Traut et al. [27] with modifications described in Dalíková et al. [5]. Female and male gDNAs were fluorescently labelled using an improved nick translation protocol based on Kato et al. [28]. The 20 µL labelling reaction contained 1000 ng of gDNA; 0.05 mM each dATP, dCTP, and dGTP; 0.01 mM dTTP; 0.02 mM labelled nucleotides with either Cy3-dUTP (male gDNA) or fluorescein-dUTP (female gDNA) (both Jena Bioscience, Jena, Germany), nick translation buffer (50 mM TrisHCl, pH 7.5, 5 mM MgCl_2_, 0.005% BSA), 10 mM β-mercaptoethanol, 20 U DNA Polymerase I (ThermoFisher Scientific, Waltham, MA, USA) and 0.005 U DNase I (ThermoFisher Scientific). The reaction was incubated at 15 °C for 2.5 h. For hybridization mixture per slide, 250 ng of each labelled probe and 25 µg of sonicated salmon sperm DNA (Sigma-Aldrich) were mixed together, precipitated, and re-dissolved in 50% deionized formamide, 10% dextran sulphate, and 2× SSC. The mixture was denaturated at 90 °C for 5 min and prehybridized at 37 °C for 1.5 h. After prehybridization, the mixture was applied on a female meiotic preparation, which had been previously treated with RNAse A (200 ng/μL; Sigma–Aldrich) in 2× SSC for 1 h at 37 °C, washed 2× 5 min in 2× SSC, and denaturated at 68 °C in 70% formamide solution in 2× SSC for 3.5 min. The slides were incubated with the hybridization mixture for 3 days at 37 °C, washed in 0.1× SSC with 1% Triton X-100 at 62 °C, and counterstained with 0.5 μg/mL DAPI in antifade based on DABCO.

### 2.4. Microscopy and Image Processing

Chromosome and polyploid nuclei preparations were examined using a Zeiss Axioplan 2 microscope (Carl Zeiss, Jena, Germany) equipped with appropriate fluorescence filter sets and a monochrome CCD camera XM10 (Olympus Europa Holding, Hamburg, Germany). Black-and-white images were captured with CellSens Standard software version 1.9 (Olympus). Preparations of polyploid nuclei were investigated using light microscopy. For CGH preparations, black-and-white images were captured separately for each fluorescent dye, and the images were pseudocolored and merged using Adobe Photoshop CS6 (Adobe Systems, San Jose, CA, USA).

### 2.5. Flow Cytometry

Flow cytometry was used to estimate the genome size, and to uncover potential differences between males and females which might correspond to the absence of the W chromosome. Two bagworm species—*P. betulina* and *T. tubulosa*—were examined. *D. laichartingella* was not examined due to lack of material. For the genome size measurement, larval brain tissue was frozen in liquid nitrogen and stored at −20 °C until next use. As the internal standard, we used fresh *E. kuehniella* adult males (1/4 of head per sample) for *P. betulina*, and fresh *D. melanogaster* adult males (1/4 of head per sample) for *T. tubulosa*; it was not possible to use a single standard for both species studied due to the overlap of peaks. The entire head of the measured bagworm individuals as well as part of the head of the standard were chopped using a sharp razor blade in 500 μL of nuclei isolation buffer (0.1 M Tris-HCl pH 7.5, 2 mM MgCl_2_, 1% Triton X-100) [29]. The suspension was filtered through a 42-μm nylon mesh, the volume was adjusted with the buffer to 1 mL, and propidium iodide (PI) and RNase IIa were added, both at final concentration of 50 μg/mL. The samples were stained at least for 20 min and analyzed with a Partec CyFlow SL flow cytometer (Partec, Münster, Germany; now Sysmex) equipped with a 100 mW 532 nm (green) solid-state laser. Fluorescence intensity and SSC (side-scattered light) parameter of 10,000–30,000 particles (depending on number of peaks and amount of debris) were recorded. Data were analyzed using FlowJo 10 software (TreeStar, Inc., Ashland, OR, USA). Due to the relatively large amount of fluorescent debris, gating based on a combination of SSC and PI fluorescence signals was applied to the samples before evaluating histograms of PI fluorescence. Mean, coefficient of variation (CV), and number of nuclei were recorded for 2C peaks of both the sample and the standard.

The genome size was calculated from the ratio of the mean fluorescence of the sample and the internal standard, *E. kuehniella* (2C = 0.90 pg; [29]) or *D. melanogaster* (2C = 0.36 pg; [30]). Basic statistics (mean, standard error, standard deviation, and variation range) of the genome size were calculated for each species and sex. Genome sizes of males and females of each species were compared using the two-sample *t*-test.

## 3. Results

### 3.1. Sex Chromatin and Chromosome Number

**Figure 1:**
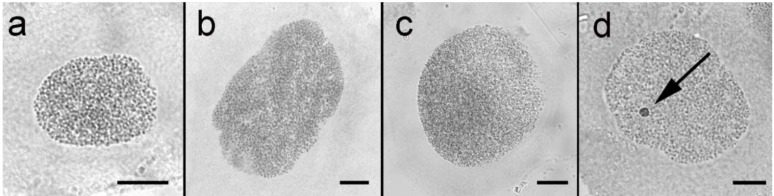
Sex chromatin assay in polyploid nuclei from Malpighian tubules of bagworm (Psychidae) female larvae. No sex chromatin was found in any species of Psychidae, namely *Proutia betulina* (**a**), *Taleporia tubulosa* (**b**), and *Diplodoma laichartingella* (**c**). In contrast, all female nuclei of the control species, *Ephestia kuehniella* (Pyralidae) showed a conspicuous sex chromatin body (arrow; (**d**)). Bar = 10 µm.

All species studied were tested for the presence of sex chromatin in the polyploid nuclei from Malpighian tubules in both males and females. Sex chromatin was absent in all specimens examined (Figure 1).

**Figure 2:**
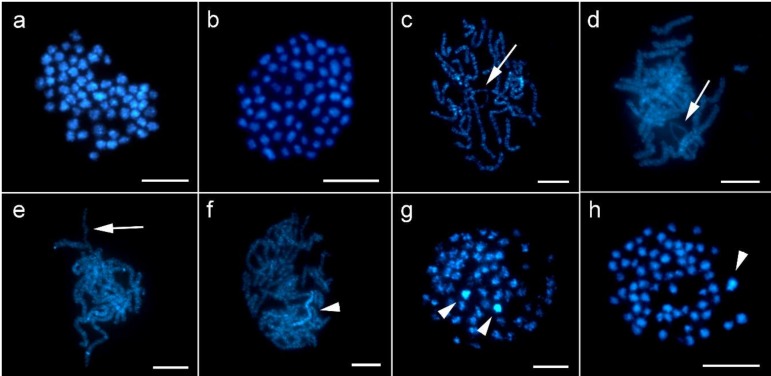
DAPI (4′,6-diamidino-2-phenylindole)-stained mitotic and meiotic chromosomes of *P. betulina*, *T. tubulosa*, and *D. laichartingella*. Different numbers of chromosomes in mitotic metaphases of *P. betulina* females with 2*n* = 61 (**a**) and males with 2*n* = 62 (**b**), and the presence of a Z-chromosome univalent (arrows) in female pachytenes of *P. betulina* (**c**), *T. tubulosa* (**d**), and *D. laichartingella* (**e**) indicate the absence of the W chromosome in these species. In *P. betulina*, a strongly heterochromatinized bivalent was observed in male pachytenes (**f**); mitotic metaphases with less condensed chromosomes showed two DAPI-highlighted chromosomes in males (**g**), and only one DAPI-highlighted chromosome in females (**h**). These heterochromatin DAPI-positive elements (arrowheads) probably represent a Z-chromosome bivalent (**f**) and Z chromosomes (**g**,**h**). Bar = 10 µm.

Chromosome preparations stained with DAPI were examined using a fluorescence microscope. We determined de novo the chromosome number in *P. betulina*, which is 2*n* = 61 in females and 2*n* = 62 in males (Figure 2a,b). Chromosome numbers of *T. tubulosa* (2*n* = 59/60 in female/male) were published by Seiler [14]. Unfortunately, we failed to determine the chromosome number in *D. laichartingella* due to lack of material.

Interestingly, in *P. betulina* two large, strongly heterochromatinized chromosomes were observed in male preparations, both as a bivalent in the pachytene stage (Figure 2f) and as two individual chromosomes in most mitotic metaphases (Figure 2g). In female preparations, however, only one such chromosome was found in most mitotic metaphases (Figure 2h). Based on the difference between male and female preparations, we presume that this conspicuous chromosome is the Z chromosome.

Importantly, a single unpaired chromosome was repeatedly observed in female pachytenes of *P. betulina*, *T. tubulosa*, and *D. laichartingella* (Figure 2c–e). Such an element was not observed in male pachytenes. This finding suggests that the unpaired element is the Z-chromosome univalent.

### 3.2. Comparative Genomic Hybridization (CGH)

**Figure 3:**
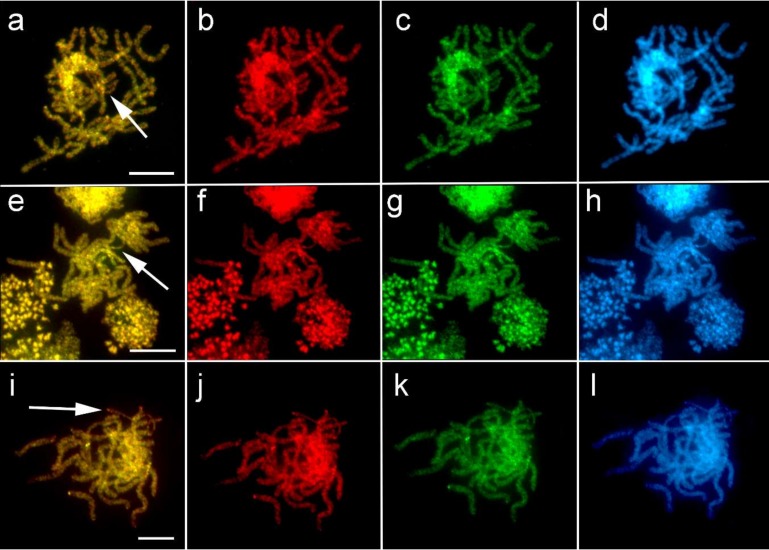
Comparative genomic hybridization (CGH) on female pachytene bivalents in *P. betulina* (**a**–**d**), *T. tubulosa* (**e**–**h**), and *D. laichartingella* (**i**–**l**). Panels **a**, **e**, **i** show merged pictures of both probes. Hybridization signals of male probes (red) are shown in panels **b**, **f** and **j**; female probes (green) in **c**, **g** and **k**. DAPI counterstaining (blue) is shown in panels **d**, **h** and **l**. Z-chromosome univalents are marked with arrows. CGH did not identify any female-specific/enriched element in pachytene complements, confirming the absence of the W chromosome. Bar = 10 µm.

To verify the absence of the W chromosome in Psychidae, we performed CGH on female meiotic preparations of *P. betulina*, *T. tubulosa*, and *D. laichartingella*. Both the female and male genomic probes hybridized evenly to all chromosomes and no chromosome was highlighted by the probes, thus supporting the absence of the W chromosome in the karyotype of these species (Figure 3a–l).

### 3.3. Flow Cytometry

**Figure 4:**
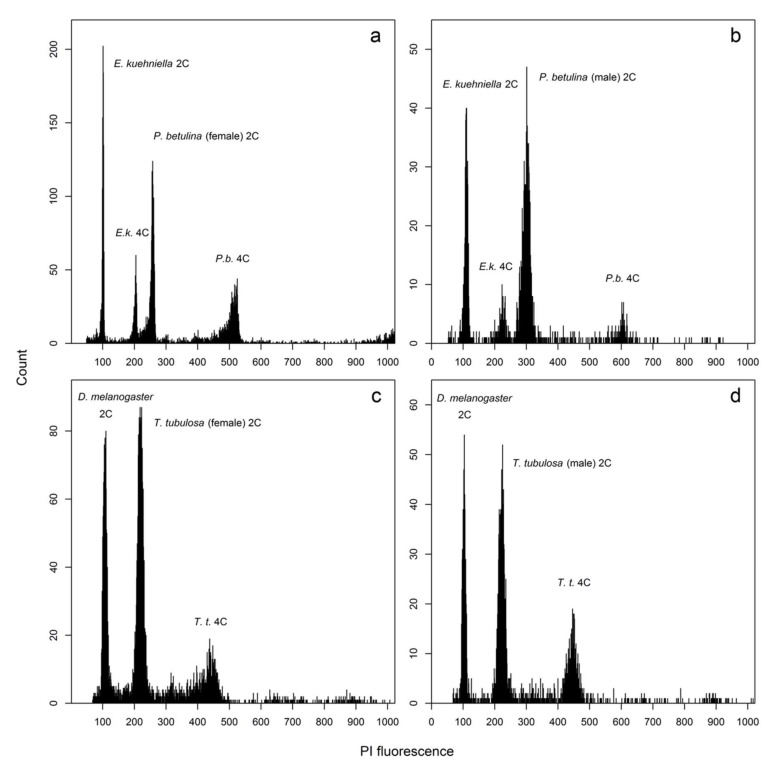
Examples of flow cytometric fluorescence histograms of Psychidae species *P. betulina* (**a**)—female, (**b**) male and *T. tubulosa* (**c**)—female, (**d**)—male). In all cases, 2C and 4C peaks are clearly visible. Peaks of the internal standards *E. kuehniella* and *Drosophila melanogaster* are denoted in a similar way. PI = propidium iodide.

To confirm the cytogenetic data, we used flow cytometry to measure the genome sizes of both sexes in *P. betulina* and *T. tubulosa*. Flow cytometric analysis of Psychidae is a challenge due to the small amount of brain tissue in the species studied and the need to use frozen material due to the short season of occurrence. However, after optimizing the amount of the internal standard and exhausting the entire volume of the sample, we were able to obtain at least three measurements of sufficient quality (clear peaks with a mean CV = 3.7% and 4.7% for the sample and the standard, respectively, and enough particles) for both sexes of both taxa. The genome size of *P. betulina* females was 2C = 2.32 ± 0.03 pg (mean ± standard error; N = 3; Figure 4a), whereas in males it was 2.45 ± 0.02 pg (N = 7; Figure 4b); on average, male genomes are bigger than female genomes by 5.6% and this difference is statistically significant (*t* = 2.876, df = 8, *p* = 0.021). The genome size of *T. tubulosa* females was 2C = 0.75 ± 0.01 pg (N = 6; Figure 4c), while the genome size of *T. tubulosa* males was 2C = 0.78 ± 0.01 pg (N = 6; Figure 4d). On average, *T. tubulosa* male genomes are bigger than female genomes by 4% and this difference is also statistically significant (*t* = 2.362, df = 10, *p* = 0.040).

## 4. Discussion

Psychidae, placed at the base of Ditrysia, have a crucial phylogenetic position for understanding the W chromosome emergence in Lepidoptera. Several cytogenetic papers on Psychidae were published by J. Seiler [14,15,16,31,32], but the methods available at the time did not allow to obtain unambiguous information about the constitution of sex chromosomes. In our study, we provide clear evidence of the W chromosome absence and a Z0/ZZ (female/male) sex chromosome system in three representatives of two major bagworm clades using a combination of classical and molecular cytogenetics and genome size measurements.

The sex chromatin assay was negative in all bagworm specimens examined, indicating the absence of the W chromosome. This method was used as indirect evidence of the W chromosome presence/absence in many lepidopteran species, which was consequently used as a basis for hypotheses about the W chromosome origin (e.g., [8,9,12,33]). However, there are some limitations, since certain chromosomal rearrangements involving the W chromosome are known to disrupt sex chromatin formation. This has been demonstrated, for example, in mutant strains of *E. kuehniella*, with radiation-induced sex chromosome rearrangements. Part of the Z chromosome was translocated on the W chromosome causing deformation and fragmentation of the sex chromatin body, while a fusion of the W chromosome with an autosome resulted in complete disintegration and disappearance of sex chromatin [22,34]. A similar sex chromatin disruption was observed in natural populations of three species of *Leptidea* wood white butterflies, in which multiple sex chromosomes originated by complex rearrangements with autosomes [35]. Recently, the absence of sex chromatin was found in females of the clouded Apollo, *Parnassius mnemosyne* (Papilionidae), although examination of sex chromosomes revealed that the W chromosome is present in this species [10]. Therefore, though the presence of sex chromatin in polyploid nuclei often corresponds to the presence of the W chromosome, its absence cannot be used as definitive proof for the absence of the W chromosome in Lepidoptera.

Due to the limitations of sex chromatin assay, it was necessary to perform a more detailed chromosome analysis of the Psychidae. Firstly, we de novo determined chromosome numbers in *P. betulina* (2*n* = 61 in females and 62 in males); the chromosome numbers in *T. tubulosa* (2*n* = 59 in females and 60 in males), were already published by Seiler [14]. This resembles data on different chromosome numbers between males and females in other species, namely *Psyche* (syn. *Fumea*) *casta* and *Luffia lapidella*, and odd chromosome number in parthenogenetic females of *Luffia fernauchtella* (all Psychinae; see Table S1 for an overview of sex chromosomes in Psychidae).

Interestingly, a Psychidae species *Apterona helix* (Oiketicinae) was reported to have equal numbers of chromosomes in females and males (reviewed in Robinson [36]). However, the original publication of Narbel [18] mentioned two possible karyotype variants that differed from each other by one chromosome. Given that the chromosome numbers in females are lower by one than in males in other Psychidae species, we believe that this could be the case for this species as well, and that the results from Narbel [18] were possibly misinterpreted by Robinson [36]. However, the karyotypes of *A. helix* should be re-analyzed to confirm their true sex chromosome constitution.

In another species of Psychidae, *Dahlica* (syn. *Solenobia*) *triquetrella*, different sex chromosome systems (Z0 and WZ) were found in local populations [16,37]. However, this “W” chromosome was described as nondisjuctional and sporadically appearing in both sexes, even in multiple copies (i.e., Z0 and WZ females and ZZ, WZZ, and WWZZ males were observed, all phenotypically normal). As initially pointed out by Robinson [36], these characteristics are more typical for B chromosomes rather than a sex chromosome. In addition, crossing experiments between diploid and tetraploid populations suggest that it is the ratio between the number of autosomal chromosome sets and Z chromosomes which determines the sex of an individual in *D. triquetrella*, not the presence of the “W” chromosome [32]. Taken together, it is not clear whether the “W” chromosome in this species is a true sex chromosome, but if so, it would certainly represent an evolutionary novelty that is not common to other Psychidae.

As mentioned above, the difference in chromosome number of males and females does not solely prove the absence of the W chromosome. However, the application of CGH did not reveal any potential female-specific or female-enriched sequences that would make the W visible, thus providing further evidence of the W chromosome absence. Consistent with this finding, the female pachytene nuclei showed a univalent not seen in male pachytenes, which was interpreted as a single Z chromosome. Taken together, we conclude that the odd chromosome number in females of studied species is the result of W chromosome absence.

Finally, we compared the genome size of males and females in *P. betulina* and *T. tubulosa*. Our results showed that the female genomes are significantly smaller than the male genomes in both species, specifically by 5.6% in *P. betulina* and by 4% in *T. tubulosa*, which supports the absence of the W chromosome in females and corresponds well with chromosome counts. The differences between male (ZZ) and female (Z0) genomes should therefore correlate with the size of the Z chromosomes, which seem to be remarkably larger than most autosomes in these species. For example, the Z chromosomes in *P. betulina* were the most conspicuous chromosomes in the karyotype. Apart from being the largest chromosomes, they were often strongly stained by DAPI, which suggests a high abundance of AT-rich repetitive sequences and partial heterochromatinization. This feature was especially noticeable in mitotic metaphases with less condensed chromosomes. In contrast, no heterochromatinization was observed in chromosomal preparations of *T. tubulosa*. Our data on the large Z chromosomes correspond to previous observations from multiple taxa that the sex chromosomes in Lepidoptera are often the largest chromosomes of the karyotype [1,21,38,39,40].

In terms of phylogeny, Psychidae form two major clades, the Arrhenophanine lineage, which includes, besides others, Naryciinae (e.g., *D. laichartingella*) and Taleporinae (e.g., *T. tubulosa*), and the Psychinae lineage, which includes Psychinae (e.g., *P. betulina*) and Oiketicinae ([41]; but see [42]). In both clades, species with odd chromosome numbers in females predominate, probably having a Z0/ZZ sex chromosome system (Table S1). Even though an exception was found in Arrhenophaninae, where some populations of *D. triquetrella* presumably have a supernumerary chromosome, available data suggests that the Z0/ZZ sex chromosome system is ancestral in Psychidae.

**Figure 5:**
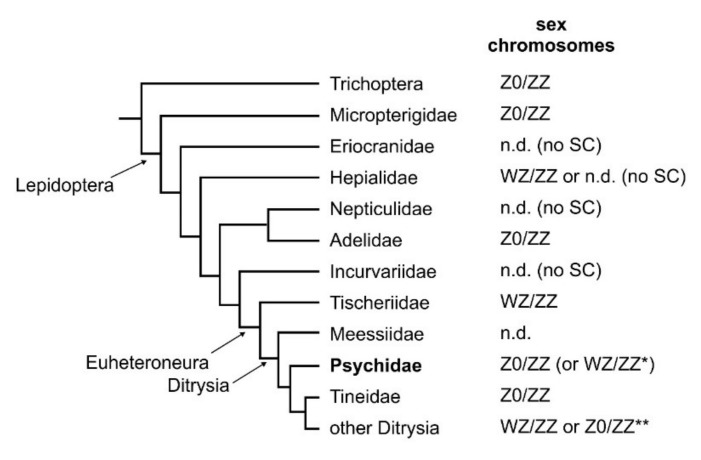
Simplified phylogenetic tree of basal Lepidoptera with known sex chromosome constitution. Records on sex chromosome status originate either from this work or from references listed in the text. In three families, only the sex chromatin (SC) assay data is available, but the actual sex chromosome status was not determined (n.d.). There are no cytogenetic data from the most basal family of Ditrysia, Meessiidae. In two species of Psychidae, namely *Dahlica triquetrella* with a supernumerary chromosome in some populations and *Apterona helix* with alleged chromosome number differences in males and females (but see the main text), the ambiguity is indicated (*). Based on the internal Psychidae phylogeny, we consider both traits to be evolutionary novelties and suggest that the ancestral state is Z0/ZZ. In other Ditrysia, the vast majority of species has a WZ/ZZ system or derived variants with multiple W and/or Z chromosomes, but some species have lost the W secondarily(**). Phylogenetic relationships are according to previous studies [43,44].

The W chromosome absence in Psychidae is important for understanding the origin of the W chromosome in Lepidoptera. It was generally believed that the W chromosome arose in the common ancestor of Euheteroneura (Tischeriidae + Ditrysia; [6,12]; see simplified phylogenetic tree of basal Lepidoptera with known sex chromosome system in Figure 5). According to this hypothesis, the presence of the W chromosome in the earliest diverging lineages of Ditrysia, i.e., Meessiidae, Psychidae, and Tineidae, was expected. However, there are no data on sex chromosomes in Meessiidae, and the W chromosome absence was recently reported in a representative of Tineidae, *T. bisselliella*, in Dalíková et al. [5], where all possible scenarios of W chromosome origin were described in detail. Briefly, based on the conserved Z chromosome synteny across Euheteroneura and the reduced chromosome number in Tischeriidae, the authors favoured (i) Z chromosome-autosome fusion in Tischeriidae and (ii) B chromosome acquisition in advanced Ditrysia (after Psychidae and Tineidae branched off) as two different ways of W chromosome birth. Both options, however, operated with the presumed absence of the W chromosome in Psychidae, which has so far been based only on odd chromosome numbers in females. Our data confirm absence of the W chromosome in Psychidae, thereby supporting the proposed hypothesis by Dalíková et al. [5].

To conclude, females of the studied species of Psychidae lack sex chromatin, have a lower chromosome number and a smaller genome size than males (not determined in *D. laichartingella*), and have a univalent in pachytene nuclei, which is absent in males. The data presented in this study thus clearly show that these species lack the W chromosome. Taken together with previous data, species with a Z0/ZZ sex chromosome system predominate in both clades of Psychidae. The putative W chromosomes, if any, have occurred infrequently and more recently in the evolution of Psychidae, suggesting an independent adoption rather than an original trait. In addition, all these findings support the hypothesis of independent origins of the W chromosomes in Euheteroneura; i.e., in Tischeriidae and in advanced Ditrysia.

## Supplementary Materials

The following are available online at https://www.mdpi.com/2073-4425/10/12/1016/s1, Table S1: Available information on chromosome numbers and sex chromosomes in Psychidae [45,46,47,48].
