# Supplementary material for: Absence of W Chromosome in Psychidae Moths and Implications for the Theory of Sex Chromosome Evolution in Lepidoptera
Source: Genes (Basel). 2019 Dec 5;10(12):1016. doi: 10.3390/genes10121016 (PMC6947638; doi:10.3390/genes10121016)
Supplement: Supplementary file 1 [file genes-10-01016-s001.pdf]

Table S1. Available information on chromosome numbers and sex chromosomes in Psychidae.

**Species**	**Subfamily**	**Reproduction mode**	**2n (♀/♂)**	**Sex chromosomes**	**Available data**	**References**
Taleporia tubulosa	Taleporiinae	sexual	59/60	Z0/ZZ	- Z univalent in female pachytene<br>- absence of sex chromatin<br>- female genome smaller than male genome	[14];<br>this study
Dahlica triquetrella syn. *Solenobia triquetrella*	Naryciinae	sexual or parthenogenetic	61/62 or 62/62	females Z0 and WZ, males ZZ, WZZ, WWZZ	- individuals with and without W do not differ in phenotype<br>- W suggested to be a B chromosome in Robinson [39]	[18,36,37,45]
Diplodoma laichartingella	Naryciinae	sexual	n.d.	Z0/ZZ	- Z univalent in female pachytene<br>- absence of sex chromatin	this study
Psyche casta syn. *Fumea casta*	Psychinae	sexual	63/64	probably Z0/ZZ	- 31 elements in female metaphase I (probably 30 bivalents, one univalent)<br>- daughter plates have either 30 or 31 chromosomes	[14]
Luffia ferchaultella	Psychinae	parthenogenetic	61 (♀ only)	probably Z0	- 30 bivalents and a single chromosome, probably Z, in female metaphase I<br>- Z called X in the paper	[17,36,46]
Luffia lapidella	Psychinae	sexual	61/62	probably Z0/ZZ	- odd chromosome number in females	[47]
Proutia betulina	Psychinae	sexual	61/62	Z0/ZZ	- Z univalent in female pachytene<br>- absence of sex chromatin<br>- female genome smaller than male genome	this study
Apterona helix	Oiketicinae	sexual or parthenogenetic	61-62	probably Z0/ZZ	- chromosome counts in original publication range from 61-62<br>- probably misinterpreted in Robinson [39]	[18,36,48]
